# Prevalence of class 1 and 2 integrons in multi-drug resistant *Escherichia coli* isolated from aquaculture water in Chaharmahal Va Bakhtiari province, Iran

**DOI:** 10.1186/s12941-015-0096-y

**Published:** 2015-07-31

**Authors:** Elahe Tajbakhsh, Faham Khamesipour, Reza Ranjbar, Ifeoma Chinyere Ugwu

**Affiliations:** Department of Microbiology, Faculty of Basic Sciences, Shahrekord Branch, Islamic Azad University, Shahrekord, Iran; Young Researchers and Elite Club, Shahrekord Branch, Islamic Azad University, Shahrekord, Iran; Molecular Biology Research Center, Baqiyatallah University of Medical Sciences, Tehran, Iran; Department of Veterinary Pathology and Microbiology, University of Nigeria, Nsukka, Enugu State Nigeria

**Keywords:** Antimicrobial resistance, Aquaculture, *E. coli*, Integrons, Iran

## Abstract

**Background:**

Integrons play important role in the spread and maintenance of antimicrobial resistance among strains of *Escherichia coli* (*E. coli*) and other species of *Enterobacteriaceae*. This study investigated the prevalence of class 1 and 2 integrons among *E. coli* strains isolated from aquaculture water of fish fields in Iran.

**Methods:**

One hundred and fifty water samples from different geographical regions in Chaharmahal Va Bakhtiari province were examined over a 2 months period. Isolation was through culture and biochemical tests. Integrons were identified through polymerase chain reaction (PCR) using oligonucleotide primers specific for class 1 and 2 integrons. Antimicrobial susceptibility testing was carried out using disc diffusion methods.

**Results:**

Eighteen percent of the water samples were positive for *E. coli*. All the strains were multi-drug resistant; 100% to ciprofloxacin, chloramphenicol, gentamycin, ampicillin and tetracycline and least resistant to imipenem (7.2%). Ten (50%) of the most resistant strains were positive for class 1 (40%) and class 2 (10%).

**Conclusions:**

*Escherichia coli* in aquaculture in Iran carried integrons class 1 and 2 which could be of public health concern since they could play a role in the spread and maintenance of antimicrobial resistance among bacterial population in the region and should be constantly monitored.

**Electronic supplementary material:**

The online version of this article (doi:10.1186/s12941-015-0096-y) contains supplementary material, which is available to authorized users.

## Background

Development of antimicrobial resistance in microorganisms has been attributed to indiscriminate use of antibiotics which poses serious public health concern worldwide [1]. Multi drug resistant strains of *Escherichia coli* (*E. coli*) can be found in both human and animal isolates worldwide [2] with multiple drug resistant non-pathogenic *E. coli* found in the intestine implicated as important reservoir of resistance genes [3]. Acquired multi-drug resistance to antimicrobial agents creates an extensive trouble in case of the management of intra and extra intestinal infections caused by *E. coli*, which are a major source of diseases, mortality and increased production costs [4]. Pathogenic *E. coli* strains could acquire resistance genes through horizontal gene transfer of mobile genetic elements like integrons which have been reported to house resistance genes. Mobile genetic elements like plasmids and transposons are known to carry integrons which can contain genes for sit-specific recombination and are capable of capturing and mobilizing gene cassettes [5, 6]. Integrons are categorized into types, the super-integrons and the antibiotic resistance integrons (ARIs) [7, 8]. Dissemination of antibiotic resistance genes among bacteria can occur by mobile genetic elements containing the ARIs (5, 7, 9). There are four classes of integrons namely classes 1, 2, 3 and 4 which are known to carry multi-drug resistance genes [7, 9, 10]. Class 1 integrons are the most widespread and have been frequently found in ESBL producing clinical isolates of *Enterobacteriaceae* [11, 12]. Class 2 integrons occur less frequently in ESBL producing *E. coli* and *Klebsiella pneumoniae* while class 3 integrons are rarely found [13]. Class 4 integrons have only been described in *Vibrio cholerae* strain [10]. The Prevalence of class 1 and 2 integrons in multi-drug resistant *E. coli* isolated from aquaculture water was investigated in this study.

## Methods

### Sample collection

A total of 150 water samples from different fish fields from different geographical regions in Chaharmahal Va Bakhtiari province, Iran were examined over a period of 2 months, from October 2013 to November 2013. Water from each fish field was collected in 1,000 mL glass bottles and was taken to the laboratory immediately for bacterial isolation. All samples were labeled to show serial number, place of water, type of water as well as time and date of collection.

### Bacterial isolation and biochemical tests for the identification of *E. coli*

Examination of the water samples was completed within 24 h after collection using Standard Total Coliform Multiple-Tube (MPN) Fermentation Techniques. After determination of MPN the tubes showing growth were inoculated onto MacConkey agar and positive ones subcultured on Eosin-methylene blue (EMB) plates (Merck). After 24 h incubation at 35 ± 0.5°C for 24 ± 2 h g negative microorganisms were isolated from MacConkey and EMB agar and determined at the species level using cytochrome oxidase, triple sugar iron agar, urea and indole tests as putatively *E. coli*.

### DNA extraction and polymerase chain reaction (PCR) conditions for detection of integrons

Purification of DNA directly from water samples filtered was achieved using a Genomic DNA purification kit (Fermentas, Germany) according to the manufacturer’s instructions. Oligonucleotide primers specific for the *E. coli* are presented in Additional file 1: Table S1 [13, 14].

The PCR reactions were performed in a total volume of 25 μL, including 1.5 mM MgCl_2_, 50 mM KCl, 10 mM Tris–HCl (pH 9.0), 0.1% Triton X-100, 200 μM dNTPs each (Fermentas), 50 pmoL of each of the *E. coli* specific primers, 1.5 U of Taq DNA polymerase (Fermentas), and 3 μL (40–260 ng/μL) of DNA.

Amplification reactions were carried out using a DNA thermo-cycler (Eppendorf Mastercycler 5330, Eppendorf-Nethel-Hinz GmbH, Hamburg, Germany) and are listed in Additional file 1: Table S1 [13, 14]. Amplified samples were analyzed by electrophoresis in a 1.5% agarose gel in 1× TBE buffer at 80 V for 30 min, stained with solution of Ethidium Bromide and examined under Ultra Violet illumination (Uvitec, UK). Twenty most resistant strains out of the 27 isolates were tested for presence of integrons in their genome using PCR technique described above. For each set of PCR reactions, was positive control for class 1 integrons.

### PCR conditions for detection of resistance genes

The presence of genes associated with choramphenicol [*cml*A], gentamicin [*aac*(*3*)-IIa, tetracycline-[*tet*(A), ciprofloxacin, norfloxacin and nalidixic acid resistance-(*qnrA*), and sulphonamides (*Sul 1* and *Sul 2*) was determined by PCR using specific primers are presented in Additional file 2: Table S2 [15–18].

### Antimicrobial Susceptibility Testing

Antimicrobial susceptibility tests were performed by the Kirby-Bauer disc diffusion method using Mueller–Hinton agar (HiMedia Laboratories, Mumbai, India, MV1084), according to the Clinical and Laboratory Standards Institute guidelines [19]. After incubating the inoculated plate aerobically at 37°C for 18–24 h in an aerobic atmosphere, the susceptibility of the *E. coli* isolates to 12 antimicrobial agents was determined, and the results were interpreted in accordance with interpretive criteria provided by CLSI [19]. *E. coli* ATCC 25922 was used as quality control organisms in antimicrobial susceptibility determination.

The following antibiotic disks (Padtan-Teb, Iran) were used: ceftazidime (CAZ) (30 μg), tetracycline (TE) (30 μg), chloramphenicol (C) (30 μg), imipenem (IMP) (10 μg), ciprofloxacin (CRO) (5 μg), norfloxacin (NOR) (10 μg), cefalotin (CF) (30 μg), nalidixic acid (NA) (30 μg), nitrofurantoin (FM) (300 μg), trimethoprim/sulfamethoxazole (SXT) (30 μg) and gentamicin (GM) (10 μg).

## Results

Out of the 150 water samples, 27 harboured *E. coli* after culture and biochemical tests. Hence the prevalence of *E. coli* in aquaculture was 18%.

### Antimicrobial resistance

All isolates exhibited multi-drug resistance phenotypically. They were most resistant to ciprofloxacin, chloramphenicol, gentamycin, ampicillin and tetracycline (100%) and were least resistant to imipenem (7.4%) Additional file 3: Table S3.

### Detection of integrons

**Fig. 1:**
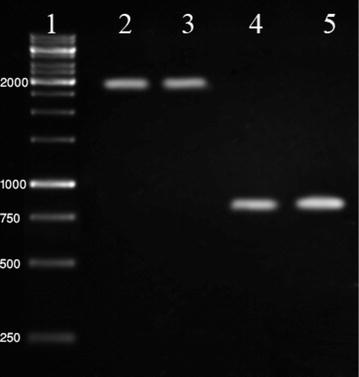
Agarose gel electrophoresis of class 1 and 2 integrons in *E. coli* strains. *Lane 1* 1,000 bp DNA Ladder, *Lane 2* positive control, *Lane 3* class 1 integrons (1,900 bp), *Lane 4 and 5* class 2 integrons (789 bp).

Out of these *E. coli* isolates, 10 (37%) carried integrons presumably in their plasmids. Eight isolates carried Class 1 integrons while 2 isolates carried class 2 integrons. Class 1 and 2 integrons were not found in any single isolate Additional file 4: Table S4. The results show in Fig. 1.

### Detection of resistance genes and integrons

Of the 27 strains that were resistant to chloramphenicol and gentamicin, 23 (85.2%) contained the *cmlA and aac*(*3*)*IIa* genes respectively while 22 (81.5%) contained *tetA* gene. *qnrA* gene that is responsible for ciprofloxacin, norfloxacin and nalidixic acid resistance was detected in one (3.7%) of the strains tested. *Sul1*gene was found in 9 (33.3%) strains while *sul 2* gene was present in 7 (25.9%) of the strains (Additional file 5: Table S5, Additional file 6: Table S6).

## Discussion

It was observed that aquaculture in Chaharmahal Va Bakhtiari province, Iran provided enabling environment for the growth and survival of microorganisms since 18% of aquaculture samples haboured *E. coli*. This could be as a result of direct contamination of the water by feacal content due to the abundance of *E. coli* in mammalian colon [20]. Fecal content could be from the intestine of the fish [21] or from manure feeding which have been reported to increase incidence of *E. coli* in aquaculture [22]. These organisms could be dangerous to the fish grown in such aquaculture thereby reducing the quantity and quality of the produce. Since *E. coli* causes disease for both fish and other mammals [23, 24], fish farmers and aquaculture attendants are at the danger of exposure to these organisms since working in aquaculture involves high degree physical contact with the water. This could lead to aquatic infections spreading to the community since *E. coli* in seafood have been implicated as a source of diarrheagenic infections [25]. To safeguard cross-infections in this instance, microbial water quality maintenance, post-harvest care, handling hygiene and adequate sanitation during processing must be adhered to strictly [25].

The *E. coli* isolated were found to be multi-drug resistant, being resistant to more than five antimicrobials commonly used for feed additives and or therapeutics; suggesting a linkage between antibiotic usage and development of resistance [26, 27]. Although these isolates were not proven to be pathogenic, their antimicrobial resistance attributes also make them dangerous as potential source of resistance dissemination among bacterial population in the region. They could constitute environmental hazard since aquaculture water when discarded could become available to other animals and could contaminate sources of water for domestic, industrial and animal husbandry use. Since these resistance attributes have been reported elsewhere to be mediated by specific genes [28] which may be transferable horizontally [29], the possibility of resistance transfer to other *E. coli* species and other bacterial organisms in a multi culture environment is high. Molecular studies on the underlying resistance mechanisms in these multi-resistant *E. coli* revealed that resistance phenotypes were mediated by several different genes. This finding suggests that the presence of these multi-resistant *E. coli* in these aquaculture water were probably due to the acquisition of resistance genes by different isolates from different sources.

This antimicrobial resistance is encoded oftentimes by integrons that occur on plasmids or that are integrated into the bacterial chromosome. We found 50% of the most resistant isolates positive for integrons, class 1 (40%) and class 2 (10%) which was similar to the report of Goldstein et al. [10] that class 1 integrons were present in approximately 46% of the isolates from the family *Enterobacteriaceae*. In that report, class 2 integrases were present only among *E. coli* and *Salmonella* isolates. Kang et al. [30] also reported 44% integrons among *E. coli* isolates in Korea. Our result was however, lower than 76% occurrence of class 1 integrons reported by van Essen-Zandbergen et al. [31] among isolates of *E. coli* in Netherlands. Again, our finding goes contrary to the report of Bass et al. [32] which previously determined that multiple-drug resistance exhibited by avian *E. coli* isolates correlated with the incidence of class 1 integrons. The presence of integrons in these isolates may not have contributed to the multi-drug resistance attributes of the isolates since the isolates without class 1 or 2 integrons exhibited nearly the same phenotypic resistance pattern with the isolates that carried class 1 or 2 integrons. However, most of the genes found in the isolates seem to be carried by class 1 integrons which is expected since class 1 type occurred more with isolates. Multi-drug resistance may also have been conferred by other genetic elements like plasmids, transposons or individual gene cassettes. Among those integron negative strains, with exception of one that was resistant to all the antimicrobials tested, all were resistant to the same class of antimicrobials and none was resistant to naldixic acid, sulfamethoxazole-trimethoprim, nitrofurantoin, imipenem and cephalotin. It seems class 2 integron do not carry the genetic basis for resistance to naldixic acid, sulfamethoxazole-trimethoprin, nitrofurantoin, and imipenem since the class 2 positive strains were susceptible to them.

Although integrons were detected in the strains of *E. coli*, their carriage did not seem to play a major role in the ability of the strains to become resistant. Similar finding was reported by Gallego and Towner [33] among clinical isolates of *Acinetobacter baumannii* in Northern Spain. However, the role of integrons in the spread and maintenance of resistance factors among the *E. coli* population and other species of the *Enterobaceriacae* cannot be overemphasized [6, 34].

## Conclusion

In conclusion, the *E. coli* strains from aquaculture in Iran carried class 1 and 2 integrons which may be contributing to the resistance of these organisms to common antimicrobials. The potential for horizontal gene transfer among bacterial population is hereby highlighted. There is therefore need for adequate monitoring to forestall perpetuation of antimicrobial resistance in the region.

## Additional files

Additional file 1:
**Table S1.** Primers used for the PCR to detect integrons. Additional file 2:
**Table S2.** Primers used for the PCR to detect Resistance genes. Additional file 3:
**Table S3.** Antimicrobial resistance profile of *E. coli* strains from aquaculture. Additional file 4:
**Table S4.** Antimicrobial resistance pattern and integron carriage among *E. coli* strains from aquaculture water. Additional file 5:
**Table S5.** Antimicrobial resistance of *E. coli* strains from aquaculture by PCR and by antibiotic disks methods. Additional file 6:
**Table 6.** Multiple drug resistance (MDR), Antimicrobial resistance gene and integron carriage among *E. coli* strains from aquaculture water.

## Abbreviations

*E. coli*: *Escherichia coli*
PCR: polymerase chain reaction
ARIs: antibiotic resistance integrons
MPN: Standard Total Coliform Multiple-Tube
EMB: Eosin-methylene blue
CLSI: Clinical and Laboratory Standards Institute
BP: base pair
CAZ: ceftazidime
TE: tetracycline
CP: chloramphenicol
IMP: imipenem
CF: ciprofloxacin
NOR: norfloxacin
C: cefalotin
NA: nalidixic acid
NF: nitrofurantoin
SXT: trimethoprim/sulfamethoxazole
GM: gentamicin
