# Supplementary material for: Prevalence of class 1 and 2 integrons in multi-drug resistant Escherichia coli isolated from aquaculture water in Chaharmahal Va Bakhtiari province, Iran
Source: Ann Clin Microbiol Antimicrob. 2015 Jul 31;14:37. doi: 10.1186/s12941-015-0096-y (PMC4521343; doi:10.1186/s12941-015-0096-y)
Supplement: Additional file 1: — Table S1. Primers used for the PCR to detect integrons. [file 12941_2015_96_MOESM1_ESM.doc]

**Table S1. Primers used for the PCR to detect integrons**

| **Gene** | **Oligoneucleotide Primer sequence (5’ - 3’)** | **PCR conditions** | **Size base pair (bp)** | **Reference** |
| --- | --- | --- | --- | --- |
| ***E. coli 16SrRNA*** | AGAGTTTGATCMTGGCTCAG  CCGTCAATTCATTTGAGTTT | 1 cycle of 2 min at 94 °C; 25 cycles of 1 min at 94 °C, 1 min at 58 °C,  1 min at 72 °C; 1 cycle of 2 min at 72 °C | 919 | [14] |
| ***Int* 1-F**  ***Int 1*-R** | GGTCAAGGATCTGGATTTCG  ACATGCGTGTAAATCATCGTC | 1 cycle of 4 min at 94 °C; 35 cycles of 1 min at 94 °C, 1 min at 62 °C,  1 min at 72 °C; 1 cycle of 8 min at 72 °C | 1900 | [13] |
| ***Int 2*-F**  ***Int 2*-R** | CACGGATATGCGACAAAAAGGT  GTAGCAAACGAGTGACGAAATG | 1 cycle of 4 min at 94 °C; 35 cycles of 1 min at 94 °C, 1 min at 62 °C,  1 min at 72 °C; 1 cycle of 8 min at 72 °C | 789 | [13] |
