# Supplementary material for: Prevalence of class 1 and 2 integrons in multi-drug resistant Escherichia coli isolated from aquaculture water in Chaharmahal Va Bakhtiari province, Iran
Source: Ann Clin Microbiol Antimicrob. 2015 Jul 31;14:37. doi: 10.1186/s12941-015-0096-y (PMC4521343; doi:10.1186/s12941-015-0096-y)
Supplement: Additional file 2: — Table S2. Primers used for the PCR to detect Resistance genes. [file 12941_2015_96_MOESM2_ESM.doc]

**Table S2: Primers used for the PCR to detect Resistance genes**

| **Gene** | **Oligoneucleotide Primer sequence (5’ - 3’)** | **PCR conditions** | **Size base pair (bp)** | **Reference** |
| --- | --- | --- | --- | --- |
| ***tetA*** | GTGAAACCCAACATACCCC  GAAGGCAAGCAGGATGTAG | 1 cycle:  94^0C^ ------------ 5 min.  30 cycle:  94^0C^ ------------ 15 s  55^0C^ ------------ 60 s  72 ^0C^ ------------ 60 s  1 cycle:  72 ^0C^ ------------ 5 min | 888 | [15] |
| ***cmlA*** | CCGCCACGGTGTTGTTGTTATC,  CACCTTGCCTGCCCATCATTAG | 1 cycle:  94^0C^ ------------ 5 min.  30 cycle:  94^0C^ ------------ 15 s  55^0C^ ------------ 60 s  72 ^0C^ ------------ 60 s  1 cycle:  72 ^0C^ ------------ 5 min | 698 | [16] |
| ***aac (3)IIa*** | CGGAAGGCAATAACGGAG  TCGAACAGGTAGCACTGAG | 1 cycle:  94^0C^ ------------ 5 min.  30 cycle:  94^0C^ ------------ 15 s  55^0C^ ------------ 60 s  72 ^0C^ ------------ 60 s  1 cycle:  72 ^0C^ ------------ 5 min | 740 | [15] |
| ***qnrA*** | ATTTCTCACGCCAGGATTTG  GATCGGCAAAGGTTAGGTCA | 1 cycle:  95 ^0C^ ------------ 5 min.  30 cycle:  95 ^0C^ ------------ 30 s  55^0C^ ------------ 60 s  72 ^0C^ ------------ 60 s | 516 | [17] |
| ***Sul 1* F** | CGGCGTGGGCTACCTGAACG  GCCGATCGCGTGAAGTTCCG | 1 cycle:  94 ^0C^ ------------ 5 min.  34 cycle:  94 ^0C^ ------------ 60 s  65^0C^ ------------ 60 s  72 ^0C^ ------------ 8 min  1 cycle:  72 ^0C^ ------------ 7 min | 433 | [18] |
| ***Sul2* R** | GCGCTCAAGGCAGATGGCATT  GCGTTTGATACCGGCACCCGT | 1 cycle:  94 ^0C^ ------------ 5 min.  34 cycle:  94 ^0C^ ------------ 60 s  65^0C^ ------------ 60 s  72 ^0C^ ------------ 8 min  1 cycle:  72 ^0C^ ------------ 7 min | 293 | [18] |
