# Supplementary material for: Prevalence of class 1 and 2 integrons in multi-drug resistant Escherichia coli isolated from aquaculture water in Chaharmahal Va Bakhtiari province, Iran
Source: Ann Clin Microbiol Antimicrob. 2015 Jul 31;14:37. doi: 10.1186/s12941-015-0096-y (PMC4521343; doi:10.1186/s12941-015-0096-y)
Supplement: Additional file 3: — Table S3. Antimicrobial resistance profile of E. coli strains from aquaculture. [file 12941_2015_96_MOESM3_ESM.doc]

**Table 3. Antimicrobial resistance profile of *E. coli* strains from aquaculture**

| **S/No.** | **Antibiotic** | **Total resistance (%)** |
| --- | --- | --- |
| **1** | Ciprofloxacin (CRO) | 27 (100%) |
| **2** | Chloramphenicol (C) | 27 (100%) |
| **3** | Gentamicin (GM) | 27 (100%) |
| **4** | Ampicillin (AM) | 27 (100%) |
| **5** | Tetracycline (TE) | 27 (100%) |
| **6** | Norfloxacin (NOR) | 24 (88.88%) |
| **7** | Ceftazidim (CAZ) | 21 (77.77%) |
| 8 | Cephalothin (CF) | 15 (55.55%) |
| **9** | Txrimethoprim-sulfamethoxazole (SXT) | 18 (66.66%) |
| **10** | Nalidixic acid (NA) | 6 (22.22%) |
| **11** | Nitrofurantoin (FM) | 6 (22.22%) |
| **12** | Imipenem (IPM) | 2 (7.40%) |
