# Supplementary material for: Prevalence of class 1 and 2 integrons in multi-drug resistant Escherichia coli isolated from aquaculture water in Chaharmahal Va Bakhtiari province, Iran
Source: Ann Clin Microbiol Antimicrob. 2015 Jul 31;14:37. doi: 10.1186/s12941-015-0096-y (PMC4521343; doi:10.1186/s12941-015-0096-y)
Supplement: Additional file 4: — Table S4. Antimicrobial resistance pattern and integron carriage among E. coli strains from aquaculture water. [file 12941_2015_96_MOESM4_ESM.doc]

**Table S4. Antimicrobial resistance pattern and integron carriage among *E. coli* strains from aquaculture water**

| **Strains** | **Resistance pattern** | **+ve for class 1 integron** | **+ve for class 2 integron** |
| --- | --- | --- | --- |
| **1** | C, CF, GM, AM, TE, NOR, CAZ, CRO, SXT, NA, FM. IMP | + | - |
| **2** | C, GM, AM, TE, CRO, NOR | + | - |
| **3** | C, NOR, GM, AM, TE, CRO | + | - |
| **4** | C, NOR, GM, AM, TE, CRO, CAZ, SXT | - | + |
| **5** | C, NOR , GM, AM, TE, CRO, CAZ, SXT | - | - |
| **6** | C, NOR, GM, AM, TE, CRO, NA | - | - |
| **7** | C, GM, NOR , SXT, AM, TE, CAZ, CRO | - | - |
| **8** | C, NOR , GM, AM, TE, CRO, SXT | + | - |
| **9** | C, CF, GM, AM, TE, CRO, CAZ | - | - |
| **10** | C, NOR, GM, AM, TE, CRO | - | - |
| **11** | C, CF, GM, AM, TE, CRO, CAZ | - | - |
| **12** | C, NOR, GM, AM, TE, CAZ, SXT, CRO | + | - |
| **13** | C, CF, GM, AM, TE, CRO, CAZ, SXT, NOR | - | - |
| **14** | C, CF, GM, AM, TE, CRO, CAZ, SXT, NOR | - | - |
| **15** | C, GM, AM, TE, NA, CRO, SXT, NOR | + | - |
| **16** | C, CF, GM, AM, TE, NOR, CAZ, CRO | + |  |
| **17** | C, NOR , GM, AM, TE CRO, CAZ, SXT | - | - |
| **18** | C, CF, AM, GM, NA, TE, NOR, CAZ, CRO | - | + |
| **19** | C, CF, GM, AM, TE, NOR, CAZ, CRO, SXT, NA, FM. IMP | - | - |
| **20** | C, NOR , GM, AM, TE, CRO, CAZ, NA | + | - |
