# Supplementary material for: Prevalence of class 1 and 2 integrons in multi-drug resistant Escherichia coli isolated from aquaculture water in Chaharmahal Va Bakhtiari province, Iran
Source: Ann Clin Microbiol Antimicrob. 2015 Jul 31;14:37. doi: 10.1186/s12941-015-0096-y (PMC4521343; doi:10.1186/s12941-015-0096-y)
Supplement: Additional file 5: — Table S5. Antimicrobial resistance of E. coli strains from aquaculture by PCR and by antibiotic disks methods. [file 12941_2015_96_MOESM5_ESM.doc]

**Table S5. Antimicrobial resistance of *E. coli* strains from aquaculture by PCR and by antibiotic disks methods**

| **Type gene** | **Antibiotics** | **Resistance by PCR** | **Resistance by antibiotic disks** | **P-value** |
| --- | --- | --- | --- | --- |
| tet A | Tetracycline | 22 (81.5%) | 27 (100%) | 0.051 |
| qnrA | Nalidixic acid | 1 (3.7%) | 6 (22.2%) | 0.1 |
| qnrA | Norfloxacin | 1 (3.7%) | 24 (88.9%) | 0.1 |
| qnrA | Ciprofloxacin | 1 (3.7%) | 27 (100%) | 0.1 |
| Sul 1 | Sulfonamides | 9 (81.5%) | 18 (100%) | 0.014 |
| Sul 2 | Sulfonamides | 7 (3.7%) | 18 (22.2%) | 0.003 |
| aac (3)Iia | Gentamicin | 23 (3.7%) | 27 (88.9%) | 0.111 |
| cmlA | Chloramphenicol | 23 (3.7%) | 27 (100%) | 0.111 |
