# Supplementary material for: Prevalence of class 1 and 2 integrons in multi-drug resistant Escherichia coli isolated from aquaculture water in Chaharmahal Va Bakhtiari province, Iran
Source: Ann Clin Microbiol Antimicrob. 2015 Jul 31;14:37. doi: 10.1186/s12941-015-0096-y (PMC4521343; doi:10.1186/s12941-015-0096-y)
Supplement: Additional file 6: — Table 6. Multiple drug resistance (MDR), Antimicrobial resistance gene and integron carriage among E. coli strains from aquaculture water. [file 12941_2015_96_MOESM6_ESM.doc]

**Table S6. Multiple drug resistance (MDR), Antimicrobial resistance gene and integron carriage among *E. coli* strains from aquaculture water**

| **Antibiotics** | **Multiple drug resistance (MDR)** | **Gene** | **Class 1 integron** | **Cass 2 integron** |
| --- | --- | --- | --- | --- |
| Chloramphenicol | 20 | cmlA (74.07%) | 8 (40%) | 2 (10%) |
| Gentamicin | 19 | aac (3)Iia (70.37%) | 7 (35%) | 2 (10%) |
| Tetracycline | 20 | tetA (74.07%) | 8 (40%) | 2 (10%) |
| Ciprofloxacin | 19 | qnrA (22.22%) | 3 (15%) | 1 (5%) |
| Norfloxacin | 6 | qnrA (22.22%) | 3 (15%) | 1 (5%) |
| Nalidixic acid | 6 | qnrA (22.22%) | 2 (10%) | 0 (0%) |
| P-value | | | 0.659 | 0.732 |
